# Supplementary material for: Associations between trajectories of obesity prevalence in English primary school children and the UK soft drinks industry levy: An interrupted time series analysis of surveillance data
Source: PLoS Med. 2023 Jan 26;20(1):e1004160. doi: 10.1371/journal.pmed.1004160 (PMC9879401; doi:10.1371/journal.pmed.1004160)
Supplement: S1 Fig — Blue solid lines indicate observed data. Dashed red lines represent counterfactuals. Counterfactual for (1) main analysis based on obesity prevalence trends from 09/2013–03/2016; (2)sensitivity analysis (a) based on obesity prevalence trends from 09/2013–12/2016; and (3)sensitivity analysis (b) based on obesity trends from 09/2013–04/2018. (DOCX) [file pmed.1004160.s002.docx]

Reforumulation increases

Study start date

Study end date

SDIL Implementation

SDIL Announcment

Main Analysis

11/2016

03/2016

Figure 1: Schematic diagram depicting overall analytical plan.

counterfactual based on trends from (09/2013 – 11/2016)

Observed data

Counterfactual based on prior observed data

11/2019

Sensitivity Analysis (a):

Sensitivity Analysis (b)

09/2013

04/2018
