# Supplementary material for: Associations between trajectories of obesity prevalence in English primary school children and the UK soft drinks industry levy: An interrupted time series analysis of surveillance data
Source: PLoS Med. 2023 Jan 26;20(1):e1004160. doi: 10.1371/journal.pmed.1004160 (PMC9879401; doi:10.1371/journal.pmed.1004160)
Supplement: S1 Text — (DOCX) [file pmed.1004160.s003.docx]

**Supplementary material A**

The model specification:

Reception children : *Yt = β_0_ + β_1_T + β_2_At + β_3_S + β_4_O + β_5_F + β_6_J*

*Year 6 children : Yt = β_0_ + β_1_T + β_2_At + β_3_S + β_4_K*

Where:

*Yt* : Average monthly obesity prevalence at month T (T=1,….,69)

*T* : Study Time (months) [1:69]

*A* : Pre (0) or post (1) announcement

*At* : Time (months) since announcement [0{29}, 1:69]

*S* : September (1) or other months (0)

*O* : October (1) or other months (0)

*J* : June (1) or other months (0)

*K* : July (1) or other months (0)

*F* : February (1) or other months (0)

And coefficients are interpreted as follows

Reception children:

*β_0_* : Intercept

*β_1_* : Trend change in obesity prevalence across time

*β_2_* : Trend change in obesity prevalence (post announcement)

*β_3_* : Seasonal variation in obesity prevalence in September

*β_4_* : Seasonal variation in obesity prevalence in October

*β_5_* : Seasonal variation in obesity prevalence in February

*β_6_* : Seasonal variation in obesity prevalence in June

Year 6 children:

*β_0_* : Intercept

*β_1_* : Trend change in obesity prevalence across time

*β_2_* : Trend change in obesity prevalence (post announcement)

*β_3_* : Seasonal variation in obesity prevalence in September

*β_4_* : Seasonal variation in obesity prevalence in July
