# Supplementary material for: Associations between trajectories of obesity prevalence in English primary school children and the UK soft drinks industry levy: An interrupted time series analysis of surveillance data
Source: PLoS Med. 2023 Jan 26;20(1):e1004160. doi: 10.1371/journal.pmed.1004160 (PMC9879401; doi:10.1371/journal.pmed.1004160)
Supplement: S1 Table — Absolute and relative changes in prevalence of obesity (95% CIs), compared to a counterfactual scenario1 based on trends prior to 8 months post-announcement, overall and by IMD in reception and year 6 children, 19 months post-implementation of UK SDIL. CI, confidence interval; IMD, index of multiple deprivation; SDIL, soft drinks industry levy. (DOCX) [file pmed.1004160.s004.docx]

|  | Total population | | Boys | | Girls | | |  |
| --- | --- | --- | --- | --- | --- | --- | --- | --- |
| Interruption: December 2016 | Percentage point change | Relative change (%) | Percentage point change | Relative change (%) | | Percentage point change | Relative change (%) | |
| Reception | | | | | | | | |
| All IMD | 0.2(0.7, -0.23) | 2.1(-2.8, 7.0) | 0.20(0.7, -0.3) | 2.0(-3.0, 7.1) | | 0.2(0.6, -0.3) | 1.92(-2.8, 6.7) | |
| IMD 1 (most deprived) | -0.5 (-0.02, -0.9) | -3.7(-7.2, -0.1) | -0.4(-0.03, -0.9) | -3.3(-6.4, -0.2) | | -0.3 (0.2, -0.7) | -2.02(-5.5, 1.5) | |
| IMD 2 | **0.4 (0.8, 0.04)** | **3.9 (0.3, 7.4)** | **0.8(1.5, 0.1)** | **6.8(0.5, 13.2)** | | 0.2(-0.3, 0.6) | 1.62(-2.8, 6.1) | |
| IMD 3 | **0.8 (0.1, 1.5)** | **8.7 (1.2, 16.2)** | 0.4 (1.1, -0.4) | 3.9(-4.0, 11.8) | | **1.0(1.6, 0.5)** | **12.0(6.1, 18.0)** | |
| IMD 4 | **0.4(0.8, 0.1)** | **5.0 (0.6, 9.4)** | 0.3(0.8, -0.2) | 3.6(-2.1, 9.3) | | 0.2(0.4, -0.01) | 2.14(-0.2, 4.4) | |
| IMD 5 (least deprived) | 0.2 (0.5, -0.2) | 2.5 (-3.0, 7.9) | 0.3(0.6, -0.1) | 3.7(-1.8, 9.2) | | 0.1 (0.5, -0.4) | 1.16(-6.2, 8.5) | |
| Year 6: |  |  |  |  | |  |  | |
| All IMD | **-0.7(-0.3, -1.1)** | **-3.21(-5.0, -1.4)** | -0.1(0.4, -0.5) | 0.3(-2.3, 1.6) | | **-1.3(-1.0, -1.7)** | **-6.9 (-8.8, -5.0)** | |
| IMD 1 | **-1.1(-0.6, -1.5)** | **-3.84(-5.5, -2.2)** | -0.1(0.4, -0.7) | -0.5(-2.3, 1.3) | | **-1.9(-1.2, -2.5)** | **-7.3(-9.7, -4.9)** | |
| IMD 2 | **-1.1(-0.7, -1.5)** | **-4.50(-6.2, -2.8)** | -0.7(-0.1, -1.3) | -2.6(-4.8, -0.4) | | **-1.6(-1.0, -2.2)** | **-7.2(-9.9, -4.4)** | |
| IMD 3 | 0.1(0.4, -0.5) | -0.39(-2.7, 1.9) | 0.03(0.7, -0.6) | 0.1(-2.8, 3.0) | | -0.2(0.3, -0.7) | -1.2(-4.1, 1.7) | |
| IMD 4 | 0.3(0.7, -0.1) | 1.71(-0.8, 4.2) | **0.6(1.2, 0.1)** | **3.3(0.6, 6.0)** | | 0.01(0.5, -0.5) | 0.1(-3.5, 3.4) | |
| IMD 5 | 0.2(0.6, -0.2) | 1.65(-0.1, 4.5) | **1.5(2.1, 0.8)** | **9.5(5.1, 13.9)** | | **-0.9(-0.5, -1.4)** | **-7.1(-10.5, -3.7)** | |

Table S1: Absolute and relative changes in prevalence of obesity (95% confidence intervals), compared to a counterfactual scenario^1^ based on trends prior to eight months post-announcement, overall and by Index of multiple deprivation in reception and year 6 children, 19 months post-implementation of UK SDIL

^1^estimated from trends within the period September 2013 to November 2016
