# Supplementary material for: Associations between trajectories of obesity prevalence in English primary school children and the UK soft drinks industry levy: An interrupted time series analysis of surveillance data
Source: PLoS Med. 2023 Jan 26;20(1):e1004160. doi: 10.1371/journal.pmed.1004160 (PMC9879401; doi:10.1371/journal.pmed.1004160)
Supplement: S2 Table — Absolute and relative changes in prevalence of obesity (95% CIs), compared to a counterfactual scenario1 based on pre-SDIL implementation trends, overall and by IMD in reception and year 6 children, 19 months post-implementation of UK SDIL. CI, confidence interval; IMD, index of multiple deprivation; SDIL, soft drinks industry levy. (DOCX) [file pmed.1004160.s005.docx]

|  | Total population | | Boys | | Girls | |
| --- | --- | --- | --- | --- | --- | --- |
| Interruption: April 2018 | Percentage point change | Relative change (%) | Percentage point change | Relative change (%) | Percentage point change | Relative change (%) |
| Reception | | | | | | |
| All IMD | **0.7(1.3, 0.1)** | **7.1(0.8, 13.4)** | **0.7(1.2, 0.1)** | **6.7(1.1, 12.4)** | **0.68(1.25, 0.10)** | **7.41(1.1, 13.7)** |
| IMD 1 (most deprived) | **0.6(1.2, 0.04)** | **5.3(0.3, 10.2)** | 0.4(0.9, -0.2) | 2.9(-1.4, 7.2) | **0.72(1.32, 0.11)** | **6.14(1.0, 11.3)** |
| IMD 2 | 0.4(0.9, -0.1) | 3.9(-0.7, 8.5) | **1.0(1.8, 0.1)** | **8.6(1.0, 16.3)** | 0.5(1.12, -0.12) | 4.74(-1.2, 10.7) |
| IMD 3 | **1.4(2.2, 0.6)** | **15.7(7.1, 24.3)** | **1.3(2.3, 0.3)** | **14.4(3.7, 25.1)** | **1.25(1.93,0.58)** | **14.16, (6.6, 21.7)** |
| IMD 4 | 0.4(0.9, -0.02) | 5.0(-0.24, 10.3) | 0.4(1.1, -0.2) | 5.3 (-2.3, 12.8) | 0.21(0.54, -0.12) | 2.54(-1.5, 6.6) |
| IMD 5 (least deprived) | -0.3(0.2, -0.8) | -4.3(-11.1, 2.5) | -0.1(0.4, -0.6) | -1.3(-8.2, 5.6) | -0.56(0.05, -1.17) | 8.13(-17.0, 0.8) |
| Year 6 | | | | | | |
| All IMD | 0.1(0.6, -0.4) | 0.4(-2.1, 2.8) | 0.2(0.8, -0.4) | 0.7(-1.8, 3.3) | -0.03(0.5, -0.5) | -0.2 (-2.9, 2.6) |
| IMD 1 | -0.2(0.5, -0.8) | -0.6(-2.9, 1.7) | -0.7(0.03, -1.4) | -2.2(-4.6, 0.1) | 0.7(1.6, -0.1) | 3.2(-0.4, 6.7) |
| IMD 2 | 0.1(0.6, -0.5) | 0.3(-2.0, 2.7) | 0.5(1.3, -0.3) | 2.1(-1.0, 5.1) | **-0.8(-0.4, -1.2)** | **-3.8(-5.7, -2.0)** |
| IMD 3 | 0.6(1.2, -0.04) | 2.9(-0.2, 6.1) | 0.9(2.4, -0.5) | 4.3(-2.3, 10.8) | **0.8(1.5, 0.1)** | **4.7(0.7, 8.7)** |
| IMD 4 | 0.4(1.0, -0.2) | 2.4(-0.9, 5.7) | **0.7(1.4, 0.03)** | **3.8(0.2, 7.4)** | 0.2(0.9, -0.6) | 1.0(-3.6, 5.6) |
| IMD 5 | 0.3(0.8, -0.2) | 2.2(-1.5, 5.9) | 0.8(1.7, -0.1) | 5.0(-0.5, 10.5) | -0.2(0.4, -0.8) | -1.4(-6.3, 3.5) |

Table S2: Absolute and relative changes in prevalence of obesity (95% confidence intervals), compared to a counterfactual scenario^1^ based on pre-SDIL implementation trends, overall and by Index of multiple deprivation in reception and year 6 children, 19 months post-implementation of UK SDIL

^1^estimated from trends within the period September 2013 to April 2018
