# Supplementary material for: Associations between trajectories of obesity prevalence in English primary school children and the UK soft drinks industry levy: An interrupted time series analysis of surveillance data
Source: PLoS Med. 2023 Jan 26;20(1):e1004160. doi: 10.1371/journal.pmed.1004160 (PMC9879401; doi:10.1371/journal.pmed.1004160)
Supplement: S3 Table — Absolute and relative changes in prevalence of excess weight (overweight or obesity) and 95% CIs, compared to a counterfactual scenario1, based on pre-SDIL announcement trends, overall and by IMD in reception and year 6 children, 19 months post-implementation of UK SDIL. CI, confidence interval; IMD, index of multiple deprivation; SDIL, soft drinks industry levy. (DOCX) [file pmed.1004160.s006.docx]

Table S3: Absolute and relative changes in prevalence of excess weight (overweight or obesity) and 95% confidence intervals, compared to a counterfactual scenario^1^, based on pre-SDIL announcement trends, overall and by Index of multiple deprivation in reception and year 6 children, 19 months post-implementation of UK SDIL

|  | Boys | | Girls | |
| --- | --- | --- | --- | --- |
|  | Percentage point change | Relative change (%) | Percentage point change | Relative change (%) |
| Class: Reception |  |  |  |  |
| All IMD | 0.3(0.9, -0.5) | 1.2(-1.8, 4.2) | 0.8(1.9, -0.2) | 3.8(-1.1, 8.7) |
| IMD 1 (most deprived) | 0.6(1.6, -0.4) | 2.4(-1.6, 6.3) | **-1.6(-1.1, -2.1)** | **-6.0(-8.0, -4.0)** |
| IMD 2 | **3.3(4.7, 1.9)** | **15.0(8.7, 21.3)** | 0.5(1.9, -1.0) | 2.0(-4., 8.2) |
| IMD 3 | 0.3(2.0, -1.5) | 1.3(-6.3, 8.8) | 2.2(3.3, 1.2) | 10.6(5.6, 15.6) |
| IMD 4 | 1.1(2.4, -0.1) | 5.5(-0.7, 11.7) | 1.9(2.9, 1.0) | 9.6(4.9, 14.4) |
| IMD 5 (least deprived) | 0.01(0.6, -0.6) | 0.04(-3.4, 3.5) | 1.5(3.1, -0.1) | 8.9(-0.5, 18.2) |
| Class: Year 6 |  |  |  |  |
| All IMD | 0.1(0.8, -0.7) | 0.1(-1.9, 2.2) | **-1.7(-1.0, -2.4)** | **-5.0(-6.9, -3.0)** |
| IMD 1 | -0.4(0.5, -1.2) | -0.8(-2.8, 1.2) | **-3.0(-2.3, -3.6)** | **-7.1(-8.7, -5.5)** |
| IMD 2 | -0.6(0.3, -1.5) | -1.5(-3.7, 0.6) | **-1.8(-0.8, -2.9)** | **-4.9(-7.7, -2.1)** |
| IMD 3 | -0.02(1.1, -1.2) | -0.1 (-3.2, 3.1) | -0.5(0.7, -1.7) | -1.5(-5.1, 2.1) |
| IMD 4 | **1.6(2.4, 0.7)** | **4.7(2.1, 7.4)** | -0.3(0.7, -1.4) | -1.2(-4.7, 2.4) |
| IMD 5 | **1.5(2.6, 0.4)** | **5.1(1.3, 8.8)** | -0.6(0.2, -1.4) | -2.3(-5.4, 0.7) |

^1^estimated from trends within the period September 2013 to March 2016
