# Supplementary material for: Associations between trajectories of obesity prevalence in English primary school children and the UK soft drinks industry levy: An interrupted time series analysis of surveillance data
Source: PLoS Med. 2023 Jan 26;20(1):e1004160. doi: 10.1371/journal.pmed.1004160 (PMC9879401; doi:10.1371/journal.pmed.1004160)
Supplement: Attachment — Submitted filename: rogers.pdf [file pmed.1004160.s007.pdf]

This paper uses existing datasets to track the influence of legislative changes on childhood obesity in England.

I was asked for a statistical report and I interpret that to include all aspects of the design and conduct of the study. The version I received did not have page numbers. My numbering has the abstract starting on page 2

Points of detail

Page 3 On the face of it the study reported here does not seem to be a clinical trial so why was it registered as such?

Page 5 I suppose it is obvious why August is excluded in England but I would not know what the equivalent month is in other countries so perhaps clarify? I had to double check to confirm it is not the same in Scotland for instance.

Page 7 Just for the record formal testing of the deprivation gradient is not needed with these numbers and a clear monotonic pattern.

Page 8 The rather strange pattern of spikes in the figures needs explaining. I appreciate is not germane to the authors' main contention but the fact that they are not consistently up or down between quintile categories, age groups or sexes is going to make the reader puzzle.

Page 23 The advertised grey bands do not look that grey to me and are quite hard to distinguish but this may not be a major concern.

Page 25 The y–axis for all boys seems to have got shifted.

Page 26 and 27 I wonder whether the caption should mention somehow that these are not on the same scale as the older age groups?

Page 28 I think this is a useful addition. With interrupted time series studies it can be hard to be clear about what happened when.

Points of more substance

The months

The authors have fitted their models with month dummies as outlined on page 6.

- Why are these different for the two age bands? Do they explain my issues with the plot spikes?

- Were these chosen based on theory or just to get a better fit? Normally I would deprecate atheoretical inclusion of covariates in the models but I can see that in this case it could be justified. I think some information about this could be included in the methods.

Interpretation

The authors have found quite a mixture of effects. I have two comments about their interpretations.

- Given that the deprivation categories do not all show the same effect is it wise to interpret an overall effect?
- The abstract spends most of its space on discussing the older age group which might lead the reader to downplay the fact that the younger children seem to have behaved differently.

I have no problems with putting all this information into the public arena but despite the authors' valiant attempts the conclusion I would draw is that we still do not really know what the mechanism underlying the changes is.

Summary

Mostly for clarification.

Michael Dewey
