# Supplementary material for: Associations between trajectories of obesity prevalence in English primary school children and the UK soft drinks industry levy: An interrupted time series analysis of surveillance data
Source: PLoS Med. 2023 Jan 26;20(1):e1004160. doi: 10.1371/journal.pmed.1004160 (PMC9879401; doi:10.1371/journal.pmed.1004160)
Supplement: Attachment — Submitted filename: year6-table2 (2).pdf [file pmed.1004160.s009.pdf]

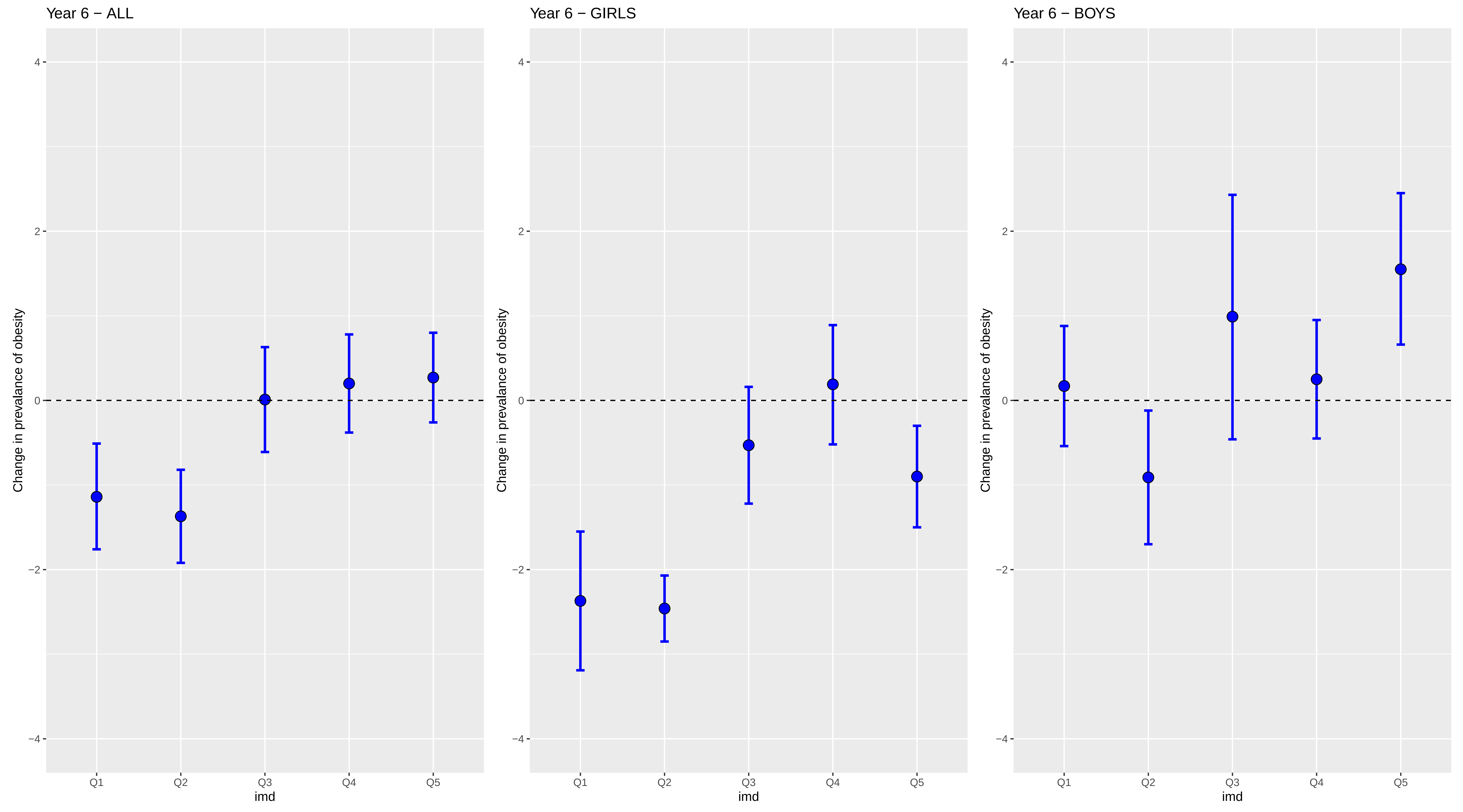
Year 6 − ALL
Year 6 − GIRLS
Year 6 − BOYS
Change in prevalance of obesity
4
2
0
−2
−4
Q1
Q2
Q3
Q4
Q5
imd
