# Supplementary material for: Associations between trajectories of obesity prevalence in English primary school children and the UK soft drinks industry levy: An interrupted time series analysis of surveillance data
Source: PLoS Med. 2023 Jan 26;20(1):e1004160. doi: 10.1371/journal.pmed.1004160 (PMC9879401; doi:10.1371/journal.pmed.1004160)
Supplement: Attachment — Submitted filename: response to reviewers_only.doc [file pmed.1004160.s008.doc]

**Response to Reviewers:**

**Reviewer #1:**

*This paper uses existing datasets to track the influence of legislative changes on childhood obesity in England. I was asked for a statistical report and I interpret that to include all aspects of the design and conduct of the study. The version I received did not have page numbers. My numbering has the abstract starting on page 2*

**Our response:** We thank reviewer 1 for the detailed and helpful suggestions and comments on our paper. The resulting revisions have improved the clarity and quality of the manuscript. We’ve added page and line numbering to facilitate the review process.

Points of detail

*Page 3 On the face of it the study reported here does not seem to be a clinical trial so why was it registered as such?*

**Our response:** Whilst ISRCTN started as a trial registry and may be primarily known as such, it has now expanded to accept “all studies involving human subjects or populations with outcome measures assessing effects on human health and well-being”. We registered the wider evaluation, of which this paper is a part, to reflect good practice principles of prospective registration and open science. Registration with ISRCTN was also a requirement of our funder, the National Institute of Health Research.

*Page 5 I suppose it is obvious why August is excluded in England but I would not know what the equivalent month is in other countries so perhaps clarify? I had to double check to confirm it is not the same in Scotland for instance.*

**Our response:** The National Child Measurement Programme collects measures on the height and weight of children in England throughout the academic school year when schools are in session. In England, schools are not in session in August – the long summer holiday runs from late July to early September. In other countries, whether there are equivalent months and what they are would depend on when schools are in session. We have clarified this point in the methods section (see lines 157-160).

*Page 7 Just for the record formal testing of the deprivation gradient is not needed with these numbers and a clear monotonic pattern.*

**Our response:** We thank reviewer 1 for confirming our current methodological decision not to include formal testing of the deprivation gradient.

*Page 8 The rather strange pattern of spikes in the figures needs explaining. I appreciate is not germane to the authors’ main contention but the fact that they are not consistently up or down between quintile categories, age groups or sexes is going to make the reader puzzle.*

**Our response:** We thank reviewer 1 for pointing out the need for more clarity about the spikes. While our main analysis relates to examination of the difference between observed and counterfactual models at time point in November the presence of the spikes is an interesting part of the story. It has been documented in the literature that children (especially those who are living with overweight or obesity) tend to gain more weight over the summer holiday months (see report: Variation in children’s BMI by month of measurement. Analysis of the 2008/09 National Child Measurement Programme dataset). We observe this same pattern with highest prevalence of obesity occurring in September / October. Although there were some months (July, September) where a smaller proportion of children were measured this still equates to over 6,000 child measurements in each school year for the month with the fewest measurements taken (September). NCMP report that it is therefore possible to conduct robust analysis of the NCMP dataset by month of measurement. We have now included the following sentences to clarify the spikes (see lines 369 – 374) “Consistent with these observations our statistical models and ITS graphs reveal spikes in obesity prevalence in the months following the summer holidays (September in reception and year 6 children and October in children in reception) and dips in other months (e.g.: in June and July) in some subgroups. These require further investigation which could contribute to understanding of seasonal variations in childhood obesity.”

*Page 23 The advertised grey bands do not look that grey to me and are quite hard to distinguish but this may not be a major concern.*

**Our response:** We thank reviewer 1 for highlighting the grey 95% confidence interval bands. We will be happy to follow the recommendations for final formatting (including colour guidelines) from PLOS medicine in due course.

*Page 25 The y–axis for all boys seems to have got shifted.*

**Our response:** Thank you for spotting that the y-axis for all boys were scaled slightly differently; we have re-adjusted these, so all y-axes are aligned.

*Page 26 and 27 I wonder whether the caption should mention somehow that these are not on the same scale as the older age groups?*

**Our response:** We thank the reviewer for this helpful suggestion about the scales which we have now included in the figure legend.

*Page 28 I think this is a useful addition. With interrupted time series studies it can be hard to be clear about what happened when*.

**Our response:** We are pleased that reviewer 1 thinks the additional interruption points included in our sensitivity analyses are useful to the reader.

Points of more substance

*The months The authors have fitted their models with month dummies as outlined on page 6. Why are these different for the two age bands? Do they explain my issues with the plot spikes? Were these chosen based on theory or just to get a better fit? Normally I would deprecate atheoretical inclusion of covariates in the models but I can see that in this case it could be justified. I think some information about this could be included in the methods.*

**Our response**: We thank the reviewer for pointing out the need for more clarity about use of the dummy months. Our decision to use dummy months was based on both evidence from the literature and improving the fit of the models. The inclusion of each month sequentially in our models for year 6 and reception children was tested to determine which months were associated with a change in the proportion of children living with obesity. As described above, it is well documented in the literature that seasonal differences are observed in the BMI of school children with weight gain typically occurring during school summer holidays especially in children living with overweight or obesity. Models for year 6 and reception age children were examined separately, partly because reception age children in England typically start school full-time, a few weeks after older children have returned. For both age groups we observed an increase in the proportion of children living with obesity in the months following the summer holidays (September in reception/ year 6 children and October in Reception children). We have added text to the methods to clarify this point (lines 191-194).

Interpretation

*The authors have found quite a mixture of effects. I have two comments about their interpretations.*

*Given that the deprivation categories do not all show the same effect is it wise to interpret an overall effect?*

**Our response:** We agree with reviewer 1 that the most important findings of the analysis relate to the specific subgroup results. Since the overall finding is not the focus of the manuscript, we have revised the abstract to delete the following line “In 10-11 year old children there was an overall absolute reduction in obesity prevalence (defined as >95^th^ centile on the UK90 growth charts) of 0.8% (95%CI: 0.3, 1.3)”.

*The abstract spends most of its space on discussing the older age group which might lead the reader to downplay the fact that the younger children seem to have behaved differently.*

**Our response:** We thank reviewer 1 for pointing out the need to include more results in the abstract in relation to how obesity prevalence in reception age children is associated with the UK SDIL. We have now included the following text with point estimates and 95% confidence intervals: “In 4-5 year old children, relative to the counterfactual, there were no overall changes in obesity prevalence in boys (0.5PP (95%CI:1.0, -0.1)) or girls (0.2PP(95%CI:0.8, -0.3)).”

*I have no problems with putting all this information into the public arena but despite the authors’ valiant attempts the conclusion I would draw is that we still do not really know what the mechanism underlying the changes is.*

**Our response:** We thank the reviewer for their support for our decision to publish these findings. Given this is an observational study, it is hard to draw definitive conclusions, particularly on mechanisms. This paper is part of a much wider evaluation of the SDIL which is exploring a range of outcomes and mechanisms of the SDIL.

**Reviewer #2: Overview and general recommendation:**

*This study estimates trajectories in the prevalence of obesity for children at ages 4-5 years and 10-11 years 19 months after the implementation of the sugar-sweetened beverages (SSBs) tax in the UK. It describes these trajectories overall, by sex and deprivation using an interrupted time series approach or ITSA. It is one of the first to examine changes in unhealthy weight among children in relation to the SSBs tax. Except for one study in Mexico, previous evidence was largely based on microsimulations or was linking relatively small changes in SSBs prices to health outcomes. Overall, this paper is clearly written, uses a valid empirical approach and research design given the setting, novel data, and presents and interprets the results well. Below are some comments that I hope will help improve it.*

**Our response:** We would like to thank reviewer 2 for conducting a very helpful and thorough review of our paper. We believe the changes requested by reviewer 2 have substantially improved the manuscript. We are also delighted that the reviewer found the paper clearly written and approved of the methodological approaches used.

*1. I suggest describing SSBs tax in more detail. While the tax is briefly described in "levels", it would be helpful to have a better understanding what it means in terms of %. How does it compare to the Mexican SSBs tax? And to sales SSBs taxes in the US? How is it similar/different? Why was it designed as a function of sugar content? Was it levied on manufacturers? What was the pass-through to consumer prices or is there another mechanism at play? Are there any previous studies estimating SSBs demand elasticities (overall, by sex and deprivation) in the UK? All these things will help the reader understand better why SSBs tax might be working and how.*

**Our response:** We thank reviewer 2 for making a useful suggestion to include some background of how different SSB taxes are structured. We have revised the introduction to include details of different SSB tax structures and how the UK SDIL differs. We include the following paragraph in the introduction (lines 91 – 99) “Over 50 jurisdictions have implemented taxes on soft drinks, although they differ in terms of how much tax is passed through to the consumer, the structure of the tax (including banded structure [22] and taxes levied in terms of volume sold[23] or as a proportion of the price [24]) and types of soft drink targeted). In March 2016, the UK government proposed a number of strategies, including a soft drinks industry levy (SDIL) on manufacturers, importers and bottlers of SSBs to reduce prevalence of obesity in childhood [25]. The two-tier SDIL, implemented in April 2018, differed from most other tax structures in that it was designed to incentivise manufacturers to reformulate soft drinks and thus move to a lower tax tier”

*2. To this end, the Introduction Is heavily focused on describing historic obesity trends in the UK. I suggest adding information on sugar as well as SSBs intake because that is essentially what SSBs tax is targeting. What is the consumption among children of interest overall, by sex and deprivation? You describe obesity rates, but what is important to understand are baselines of the mechanism through which SSBs tax is likely to operate.*

**Our response:** We are grateful to reviewer 2 for making a helpful suggestion to include some information around the demographic patterns relating to intake of sugar in children. We have amended the early part of our introduction with the following sentences (lines 77-83) “Children and adolescents in the United Kingdom are particularly high consumers of added sugars[4] with consumption typically peaking at approximately 70g/day in late adolescence, equivalent to over twice the recommended maximum intake of 30g[5]. SSBs are the primary source of free-sugar in the diets of children and are associated with weight gain, obesity and fatness in children [6–8]. Demographic patterns of SSB and added-sugar consumption mirror each other with highest consumption in older children [5,9], boys [9,10] and children from lower socioeconomic groups[11–13]”.

*3. Briefly explain what the evidence on tax & reformulation says exactly, so the reader doesn't have to go to reference [20] to find out.*

**Our response:** We have included some key information from the manuscript by Scarborough et al, 2020, which describes associations between the UK SDIL on overall price increases, the change in content of sugar in soft drinks and the finding that the levy was only partially passed on to the consumer. We include the following sentences (see lines 107 – 114) “Evidence suggests that the UK SDIL led to substantial reformulation of the UK soft drinks market. The percentage of drinks containing > 5g sugar/ 100ml fell from 49% to just 15% between September 2015 and February 2019, with reformulation accelerating after announcement of the UK SDIL[28]. Overall the UK SDIL was associated with a reduction in sugar purchased from soft drinks[29]. While the price of soft drinks increased following implementation of the SDIL the levy was only partially passed on to the consumer. For example, in drinks containing between ≥ 5 to <8g of sugar per 100 ml approximately one third of the levy was passed on[28]”.

*4. Statements referring to the existing evidence (and therefore the gap that this study is filling) are not fully correct. The study published in JAMA Pediatrics that studies unhealthy weight changes among adolescents post SSBs tax in Mexico does not use subjective measures, but objectively measured clinical data. Also, in Mexico, SSBs price changes post tax varied, but on average went up about 10 %. Therefore, your rationale that evidence across studies is mixed cannot be that data relies on self-reported weight and that minimal price increases in prices of SSBs are below 5% - those studies mostly study sales SSBs taxes in the US. So, while that may be the case in most previous work studies, it is not true for the studies you are describing just above that explanation. While I understand what you are trying to say, I suggest rewriting the paragraph for correctness. Also, I suggest to more clearly explain how these papers are different from this study in particular, not only how they are different between each other. For instance, you don't link SSBs price changes to health, but do a before/after SSBs tax comparison - using a very novel and well-powered data tracking children's weight over time, which is quite hard to come by and unique, so I suggest stressing that. I also suggest you also look at the paper by Aguilar et al. (2021) for before/after tax comparison in the context of Mexico - I did not see this study on the reference list. You can discuss (here or in other sections) differences in approaches between studies (e.g., regression discontinuity design vs ITSA).*

**Our response:** We apologise for not being clearer in our paragraph where we attempt to provide some explanations for the different findings among the different studies. To improve the readability and clarity of our paragraph, we have now revised it and added individual references next to each explanation so that the reader is aware of which study we are referring to. For reference the following paragraph (lines 118-127) now reads “A few studies have used empirical data to estimate associations between SSB taxes and weight-related outcomes in children and adolescents and have either shown no overall association [23,34–36] or small to modest associations in specific subgroups such as low-income households [36] children with higher BMIs [36,37] or in adolescent girls but not boys[38]. Different findings from these discrete studies may be related to use of different outcome measures (in particular, one study relied on subjective measures of self-reported weight[34]), differences in change in SSB prices achieved by taxes (some were associated with small average increases in prices of SSB (<5%)[34,35]) or differences in substitutions to high calorie untaxed food[23] and drinks[23,35]”.

We also agree with reviewer 2 that we should highlight the unique data source we use and have edited the strengths and limitations section of the discussion which now includes the paragraph (see lines 350- 352) “This study makes use of a unique and well-powered on-going nationally representative sample covering over 90% of children aged 4-5 and 10-11 years in state-run primary schools over the study period and tracks the prevalence of overweight and obesity in over one million school children annually.”

Finally, we thank the reviewer for their suggestion of including in our review of the literature, the important work by Aguila et al, 2021. We have now updated our reference list and included details of this study in our introduction.

*5. I suggest including calendar month indicators instead of including only selected months by hand. Let the data decide what is needed*.

**Our response**: In the analysis we have used a data driven approach in that we tested models for goodness of fit and ran models individually to determine which months were associated with statistically significant changes in prevalence of obesity. Our statistical results further align with the published literature showing that in the months following the summer holidays there are increases in prevalence of children living with obesity. We have added some text to the methodology section to clarify (see lines 189-194), which states: “The inclusion of each month sequentially, in our models for year 6 and reception children were tested to determine which months were associated with a change in the proportion of children living with obesity. Models for year 6 and reception age children were examined separately because reception age children in England typically start school full-time, a few weeks after older children have returned. Model specifications for year 6 and reception class children are included (S1 Text).”

*6. It is not clear at what level did you cluster your standard errors. I suggest clustering them at the school level, as you have more than 17000 schools and enough students within each.*

**Our response:** Our analysis was conducted at the level of the population. We did not have access to school level data and so are unable to cluster in the way suggested. We have clarified that this was population level data in the methods.

*7. Discussion of the absolute results is confusing - you explain absolute differences in %? I believe you should state absolute changes in percentage points not percent.*

**Our response**: We thank reviewer 2 for pointing out that absolute differences should be discussed as percentage point differences. We have made this change throughout.

*8. Were differences in IMD quintiles significantly different from each other? It does not seem to be the case from looking at the confidence intervals, but please, test this formally and report whether that is the case. You can achieve this by including an interaction term in one regression - from what I understand, you estimate each regression model separately for each subgroup. Make sure to explain this clearly in the methods section as well.*

**Our response**: Regression models for the analysis were conducted separately for each IMD quintile in each case. We didn’t include interactions for these analyses because of the clear monotonic deprivation gradient. As the statistical reviewer (1) commented that they do not believe formal testing is required here, we have not added formal interaction analyses.

*9. Why did obesity prevalence increase among reception children? Report results with numbers for that too to better understand the magnitude and statistical significance of the effects. If magnitudes are small, fine, if not, these results deserve a detailed discussion, too.*

**Our response:** We thank the reviewer for suggesting we report more results on changes in obesity prevalence in reception children. Firstly we have included more information in the results section of the abstract “In reception children, relative to the counterfactual, there were no overall changes in obesity prevalence in boys (0.5PP (95%CI:1.0, -0.1)) or girls (0.2PP(95%CI:0.8, -0.3)).

We have also included estimates in the results section of the manuscript (lines 261-264) which now reads “In reception children, compared to the counterfactual there was no absolute change in obesity prevalence overall in girls (0.2PP(95%CI: 0.8, -0.3)) and boys (0.5PP (95%CI: 1.0, -0.1)). Examination by IMD and sex showed a consistent increase in prevalence of obesity, compared to the counterfactual, in the least deprived IMD groups in both girls (0.6PP(95%CI: 1.2, 0.003) (figure 3) and boys (0.6PP(95%CI: 1.1, 0.1) (figure 4) in reception class”.

*10. You carefully apply several sensitivity analyses and mention your limitation, including the potential issue of selection bias. Can you check whether selection bias is present in the data? Provide results in the appendix. For instance, do you see smaller opting out pre- post-tax in poorer areas? How would this bias your results given that these are the groups responding the most to the tax?*

**Our response:** Unfortunately, the data on specific response rates by IMD and other groupings is not available. However, as stated (lines 150-152) the overall response rate remained very high (>90%) across the study period.

*11. I think comparison of your results to those in Mexico are interesting (e.g., 1.3pp change) - however, it is important to understand how SSBs taxes compare as well. That is why including more details on this and other SSBs taxes in the Introduction (or a bigger discussion in the Discussion) will be very helpful. Also, study in Mexico finds larger effects for heavier girls at baseline - did you check for that in your study? Given that the literature has previously reported higher health elasticities for heavier individuals I suggest you check that in your data as well (overall and by sex). It could also help explaining why your estimates are larger in more deprived areas where obesity seems to be the highest.*

**Our response:** We agree that inclusion of details of other taxes has improved the overall readability of the manuscript. We have reiterated in the discussion the limitations of comparing with the study in Mexico by adding the following sentence (see lines 312-319): “We note however that the tax implemented in Mexico is not directly comparable with the UK SDIL; in Mexico; the tax had a different design aimed at increasing the price to consumers resulting in 100% of the SSB tax being passed through to consumers, equating to a 14% increase in prices[50], and importantly the tax was included as a wider package of anti-obesity measures which included charging 8% on high energy foods[23]. We note the importance of the finding that the tax in Mexico was more effective in girls who were heavier. Similar analysis was not possible here because we only had access to repeated cross sectional data which cannot be linked over time”.

*12. This study does not provide causal estimates, so I suggest that you do not refer to them as such. There is no control group (besides the projected counterfactual trend), and this is not a clinical trial. Just because something was reported previously, it does not make it causal - especially if previous literature is heavily observational. Labeling your estimates associations is perfectly fine - nothing is wrong with that, if they are robust. However, study study's research design does not allow us to call them causal.*

**Our response:** We agree that causal language is not appropriate in this observational study and have made necessary changes.

*13. In the Discussion, you mention that you "…found that reductions in sugar intake in relation to the levy were greatest in children who were likely to be higher consumers of SSBs…" This is confusing - nowhere is this mentioned in the data, analyses or results. Is that from another paper of yours? I suggest rewriting so that it is clear you did not analyze diet data in this study.*

**Our response:** Thank you for pointing out the need to clarify this sentence. We have now edited it (lines 314-316) to read “Third, we found that reductions in obesity in relation to the levy were greatest in children who were older and from the most deprived areas. Previous studies have reported the same children are more likely to be higher consumers of SSBs [5,9,11–13]. This suggests a possible dose-response gradient between consumption levels and effectiveness of the levy in reducing obesity.”

*14. Perhaps you want to include a brief discussion that these are short term effects and may increase over time as sugar may be addictive long term. So, in that sense, your estimates are a lower bound.*

**Our response:** We thank reviewer 2 for suggesting the inclusion of an important point, which is the longer-term benefits of the UK SDIL on obesity prevalence in England. We have added the following sentence in the discussion section (lines 373-374): “Furthermore, our ITS graphs reveal that in some groups there may be continued improvement in the longer-term with a widening between counterfactual and observed values in for example, year 6 girls (IMD 1,2, and 5)”.

*15. I like the figures - the break in trend is visible. I suggest you stress the break in trend more in your results discussion as this supports their robustness. I also strongly suggest writing out an equation/regression model you estimate in the appendix, and describe a coefficient of interest, along with controls included etc… Though quite straightforward, I think it will help to understand your methodology better.*

**Our response:** We thank reviewer 2 for pointing out the need to include equations that describe our regression models. These have now been added as part of the supporting information and are referred to in the methods (see lines 193-194) “Model specifications for year 6 and reception class children are included in (S1 Text)”. We have also mentioned the clear break in trends in lines 248-249.

*16. As mentioned above, in tables, include interactions to test for difference across subgroups. Otherwise, it is hard to say whether one group had a bigger effect than the other.*

**Our response:** As discussed above and reiterated by reviewer 1 there is no need to include interaction models to test across the subgroups since there is a clear monotonic gradient in deprivation subgroups.

*Reviewer #3:*

*This is a well thought-out and well written piece of work. It provides a valuable evaluation of an important public health strategy, in a population with fairly high intakes of SSBs (particularly the year 6 children), providing a good rationale for the analysis. The methods are well described and sound, and you have included sensitivity analyses with various interruption points to account for varied implementation of the SDIL. Incorporating sex and deprivation into the analyses was a useful addition and has allowed for a more thorough understanding of the impact of SDIL. The discussion draws reasonable conclusions and considers the findings in relation to existing work, including regions with a similar strategy in place e.g. Mexico. You have considered key limitations of the work and have tempered your findings by drawing attention to the relatively small, albeit significant, impact of the strategy and the need for additional strategies to tackle the obesity crisis in children. I have suggested some minor additions to the write-up below, where I would have liked some clarification or some consideration of these points.*

**Our response:** We thank reviewer 3 for pointing out the importance and rationale for our study. We appreciate the positive comments and review of our study.

*Statistical analysis:
1. Interrupted time series (ITS) analyses were conducted to assess obesity prevalence in relation to the UK SDIL in children attending primary school reception or year 6 classes, overall and by sex and index of multiple deprivation (IMD) quintile - please confirm here that the analyses related to deprivation was by school IMD rather than pupil-level IMD*

**Our response:** Deprivation was measured by IMD at the school-level rather than the individual level (see lines 156-157). We have provided some discussion on the potential limitations of this measure in the discussion section (see lines 359-363) “Socioeconomic disadvantage was assessed using an area-level indicator (IMD) of the school that each child attended, a less sensitive measure than capturing socioeconomic disadvantage at the household level. However, there is a strong correlation between school-level IMD and the proportion of pupils eligible for free school meals, a measure of the number of children attending a school with a low household income,[65] suggesting that the measure used here is a suitable proxy measure of household deprivation.”

*2. models included adjustment for months that showed significant changes in obesity prevalence (September, October, June and February for reception class and September and July for year 6) - was this for every year within the period? (Are you assuming that these significant changes occur/are the same every year or did you only select the months and years where significant changes were observed?)*

**Our response:** We modelled different scenarios where each month (11 months: September to July) was included in the model in turn. We considered goodness of fit and included any months in the final model that were statistically significant after testing each in turn. Months were considered and modelled across the entire study period rather than in a single year. The following statement has been added to the methods for clarity (see lines 189-194) “The inclusion of each month sequentially in our models for year 6 and reception children were tested to determine which months were associated with a change in the proportion of children living with obesity. Models for year 6 and reception age children were examined separately because reception age children in England typically start school full-time, a few weeks after older children have returned. Model specifications for year 6 and reception class children are included (S1 Text).”

*Discussion:
1. Are the study team aware of any:
* pre-emptive reformulation within major drinks brand prior to announcement, that may have provided an additional interruption point, in addition to the two already explored?*

**Our response:** Published evidence (see Scarborough et al, 2020) points to significant reformulation occurring some months following the announcement of the UK SDIL. In sensitivity analysis 1 (described in p8), we selected two additional interruption points (8 months following the announcement when reformulation appear to be increasing rapidly and at implementation) to support our main analysis.

** any other obesity prevention strategies over this time period, that correlate with the interruption points, that may have impacted on overweight/obesity trends, e.g. introduction of revised school food standards, roll out of universal infant free school meals? Have these other strategies been considered in your thinking around the attribution of these changes in obesity to the SDIL?*

**Our response:** We thank reviewer 3 for raising an important point about attributing changes in the outcomes of interest to the SDIL and consideration of other events. In terms of the wider UK sugar reduction strategy, evidence so far suggests that the strategy has led to minimal changes in purchasing of sugar beyond the effects of the SDIL. Whilst there is ongoing obesity policy work in the UK, we are not aware of any policies that were introduced coincident with the SDIL. For example, universal infant free school meals began in 2014; school food standards became mandatory in Jan 2015; while SDIL was announced in 2016 and implemented in 2018.
